# Current Evidence on Virtual Reality–Based Interventions for the Treatment of Mental Disorders

**DOI:** 10.1192/j.eurpsy.2023.1118

**Published:** 2023-07-19

**Authors:** N. Tsamitros

**Affiliations:** Psychiatry and Psychotherapy, Psychiatric University Clinic of Charité in St. Hedwig Hospital, Charité - Universitätsmedizin Berlin, Berlin, Germany

## Abstract

**Introduction:**

Virtual reality (VR) enables immersion in an interactive digital world with realistic experiences, that can be applied for controlled and personalized interventions.

**Objectives:**

This presentation aims in summerizing the current research on VR in the treatment of mental disorders.

**Methods:**

Selective literature search in PubMed and Google Scholar.

**Results:**

An increasing number of publications report the therapeutic application of VR for the treatment of mental disorders. Most VR applications are based on established therapy approaches, such as exposure therapy. According to meta-analytic data, virtual exposure therapy (VRET) for specific phobia and agoraphobia with panic disorder is as effective as traditional in vivo exposure therapy. VRET for the treatment of social phobia is significantly more effective than waitlist and placebo control groups with, however, currently inconsistent metanalytic results when compared to in vivo exposure therapy. VRET for the treatment of posttraumatic stress disorder (PTSD) is similar in effectiveness compared to active psychotherapy. For psychosis, positive results have been reported for the VR-based treatment of auditory verbal hallucinations. For patients with a substance use disorder, VR can induce craving, with still unverified diagnostic and therapeutic relevance.

**Conclusions:**

VRET can broaden the psychotherapy options for anxiety disorders. Encouraging results of VR-based treatments for psychosis and PTSD indicate the need for further research concerning its effectiveness and safety. In the field of substance use disorders, evaluation of clinical-orientated VR applications is needed.

**Disclosure of Interest:**

N. Tsamitros Grant / Research support from: Berlin Institut of Health (BIH) through the Digital Health Accelerator Program

